# Supplementary material for: Grassland ecological compensation policy in China improves grassland quality and increases herders’ income
Source: Nat Commun. 2021 Aug 3;12:4683. doi: 10.1038/s41467-021-24942-8 (PMC8333364; doi:10.1038/s41467-021-24942-8)
Supplement: Supplementary file 1 — Supplementary Information [file 41467_2021_24942_MOESM1_ESM.pdf]

Supplementary Information

Grassland Ecological Compensation Policy in China Improves Grassland Quality and Increases Herders' Income

Lingling Hou, Fang Xia, Qihui Chen, Jikun Huang, Yong He, Nathan Rose, Scott Rozelle

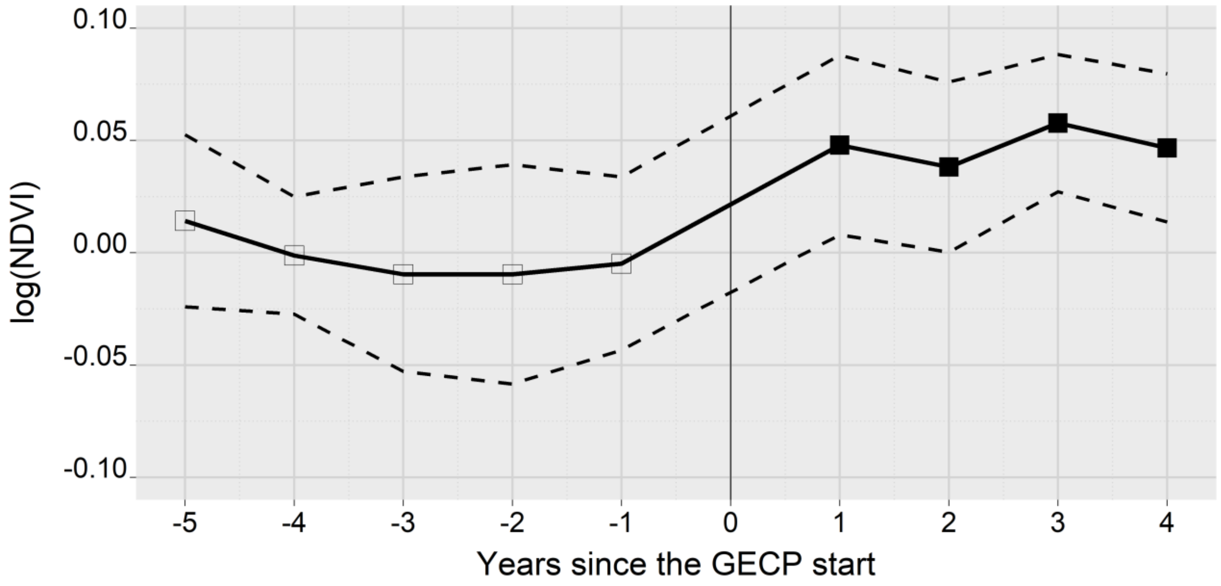

Supplementary Figure 1. Impacts of GECP on grassland quality at the county level: event study analysis

Note: Hollow squares denote estimates that are statistically insignificant at the 10% level. Solid squares denote estimates that are statistically significant at <5% level.

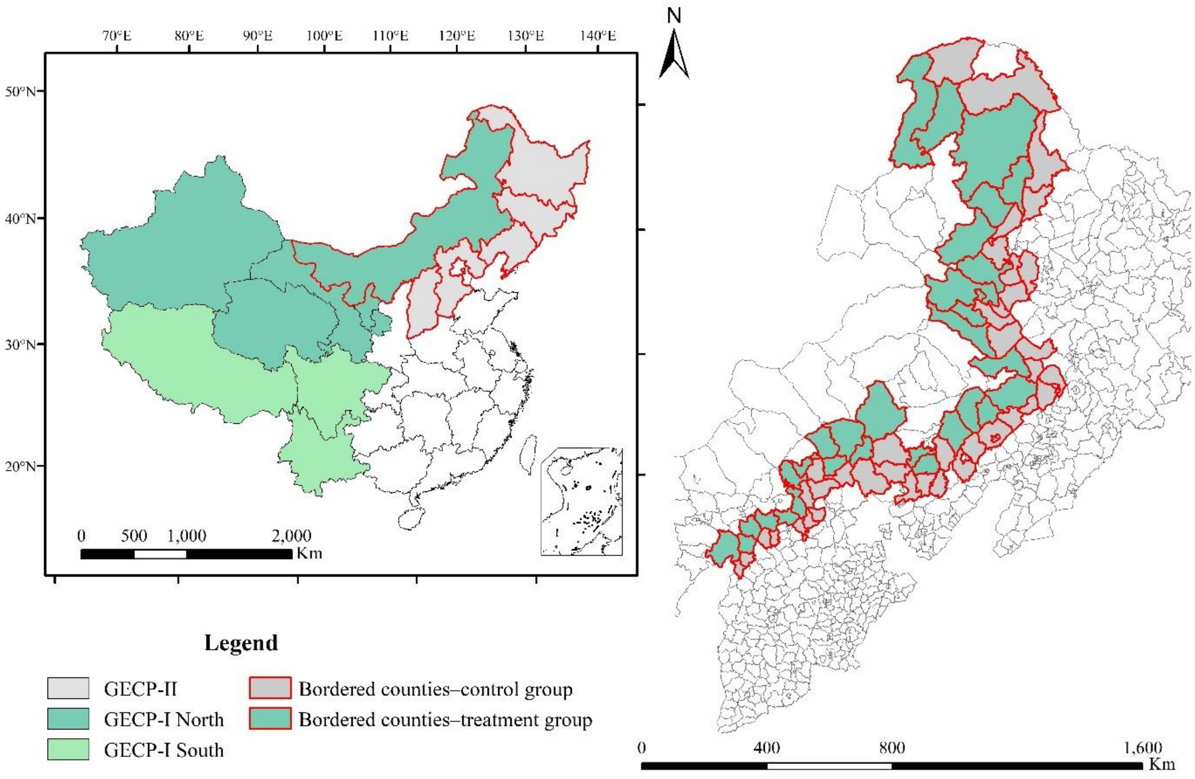

Supplementary Figure 2. GECP coverage and study area in county-level analysis

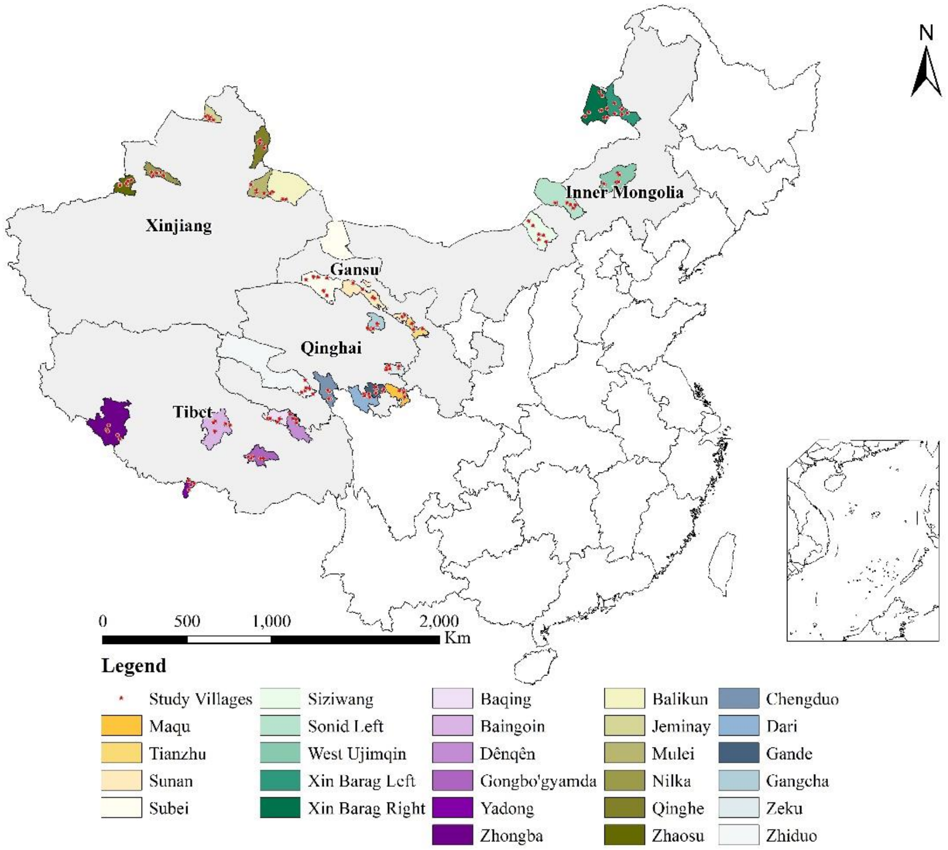

Supplementary Figure 3. Study area and sample distribution in household-level analysis

Supplementary Table 1. Distribution of grassland quality, measured by *log*(NDVI): county level

	(1)	(2)	(3)
GECP expansion	Provinces originally covered in GECP-I	Provinces added in GECP-II	Difference (= (1)–(2))
(1) 2006–2010	-0.688	-0.314	-0.374***
	[0.548]	[0.169]	(0.024)
N	2,964	1,427	
(2) 2011–2015	-0.655	-0.293	-0.362***
	[0.529]	[0.162]	(0.024)
N	2,958	1,425	
(3) Difference (= (1)–(2))	0.033***	0.021***	0.012***
	(0.000)	(0.000)	(0.001)

Note: Provinces originally covered in GECP-I include Xinjiang, Qinghai, Gansu, Ningxia, Inner Mongolia, Yunnan, Sichuan, and Tibet; provinces newly added in GECP-II include Shanxi, Hebei, Liaoning, Jilin, and Heilongjiang. Standard deviations are in brackets. Robust standard errors are clustered at the county level. A two-sided *t* test is performed for each coefficient. Exact *p*-values are in parentheses.
*$p < 0.10$, **$p < 0.05$, ***$p < 0.01$

Supplementary Table 2. Robustness checks for DID estimates of GECP impacts on *log*(NDVI) with different treatment and control groups

	(1)	(2)
Treatment group:	All counties in bordered treatment provinces (Inner Mongolia)	Bordered treatment counties (Inner Mongolia)
Control group:	All counties in bordered control provinces (N-NE provinces)	Bordered control counties (N-NE provinces)
$P \times T$	0.060***	0.030**
	(0.000)	(0.024)
Year fixed effects	Yes	Yes
County fixed effects	Yes	Yes
Climate controls	Yes	Yes
Socioeconomic controls	Yes	Yes
N	2,206	395
R^2	0.980	0.958

Note: This table provides the results from the DID approach using county-level data (Equation (1)). In column (1), the treatment group (P = 1) consists of all counties in Inner Mongolia (the only treatment province that shares its border with control provinces), and the control group (P = 0) consists of all counties in the five North and Northeastern provinces that were not covered by GECP until 2016 (i.e., Shanxi, Hebei, Liaoning, Jilin, and Heilongjiang). In Column (2), the treatment group (P = 1) consists of all counties in Inner Mongolia that share their borders with counties in Shanxi, Hebei, Liaoning, Jilin, and Heilongjiang (P = 0). The pre-program period (T = 0) is 2008–2010. The post-program period (T = 1) is 2011–2013. Year and province fixed effects are controlled for. Climate controls include monthly rainfall, temperature, and PSDI for May to October in each year. The socioeconomic control includes per-capita county fiscal income. Robust standard errors are clustered at the county level. A two-sided t test is performed for each coefficient. Exact p-values are in parentheses.

*$p < 0.10$, **$p < 0.05$, ***$p < 0.01$

Supplementary Table 3. Robustness checks for DID estimates of log(NDVI) with different post-program time periods

	(1)	(2)	(3)	(4)	(5)
Pre-program period:	2010	2010	2010	2010	2010
Post-program period:	2011	2011–2012	2011–2013	2011–2014	2011–2015
$P \times T$	0.013	0.034***	0.037***	0.047***	0.044***
	(0.188)	(0.000)	(0.000)	(0.000)	(0.000)
Year fixed effects	Yes	Yes	Yes	Yes	Yes
County fixed effects	Yes	Yes	Yes	Yes	Yes
Climate controls	Yes	Yes	Yes	Yes	Yes
Socioeconomic controls	Yes	Yes	Yes	Yes	Yes
N	1,138	1,709	2,278	2,845	3,415
R^2	0.997	0.991	0.991	0.991	0.991

Note: The treatment group ($P = 1$) includes the counties in five North and Northwestern program provinces that were covered in GECP-I, i.e., Xinjiang, Qinghai, Gansu, Ningxia, and Inner Mongolia. The control group ($P = 0$) includes the counties in five North and Northeastern provinces that were not covered by GECP-I until 2016, i.e., Shanxi, Hebei, Liaoning, Jilin, and Heilongjiang. Year and province fixed effects are controlled for. Climate controls include monthly rainfall, temperature, and PSDI for May to October in each year. The socioeconomic control includes per-capita county fiscal income. Robust standard errors are clustered at the county level. A two-tailed t test is performed for each coefficient. Exact p-values are in parentheses.

*$p < 0.10$, **$p < 0.05$, ***$p < 0.01$

Supplementary Table 4. Tests for pre-trend assumptions of fixed-effect models: household level

	(1)	(2)	(3)	(4)	(5)	(6)
Dependent variable	NDVI	Livestock	Cattle	Sheep	Supplementary feeding	Grassland rent in
Payment intensity × year 2008	0.003	0.018	0.052	0.029	-0.055	-0.004
	(0.783)	(0.721)	(0.427)	(0.600)	(0.358)	(0.736)
Payment intensity × year 2009	0.006	0.003	0.007	0.009	-0.009	0.002
	(0.644)	(0.958)	(0.913)	(0.857)	(0.881)	(0.851)
Payment intensity × year 2015	0.020**	-0.029	-0.059	0.010	0.018	0.033**
	(0.045)	(0.512)	(0.342)	(0.833)	(0.778)	(0.012)
Payment intensity × year 2016	0.017*	-0.029	-0.021	-0.022	0.007	0.030**
	(0.082)	(0.544)	(0.772)	(0.665)	(0.895)	(0.013)
Payment intensity × year 2017	-0.009	-0.073	-0.039	-0.083	0.080	0.046***
	(0.461)	(0.137)	(0.588)	(0.164)	(0.168)	(0.003)
Year fixed effects	Yes	Yes	Yes	Yes	Yes	Yes
Household fixed effects	Yes	Yes	Yes	Yes	Yes	Yes
N	2,020	2,020	2,020	2,020	2,020	2,020
R^2	0.962	0.658	0.874	0.928	0.812	0.848

Note: This table provides the results of testing the pre-trend assumptions of fixed-effect models (Equation (4)) using Qinghai and Gansu data. The dependent variable, NDVI, is log transformed. The year-end livestock and subsidy intensity are transformed using an inverse hyperbolic sine transformation to avoid taking logarithm of zeros, following $\ln(y+(y^2+1)^{1/2})$. Household-, village-, and township-level time-variant variables are also controlled for. Household-level controls include quantity of labor used in raising livestock, operated farm size, share of joint operated area, total number of different plots, a dummy variable for grassland harvesting, and a dummy variable for planting crop/fodder. Village-level controls include an indicator of whether a village has local grassroots measures in place to limit grazing intensity, an indicator of whether a village has a formal government-run monitoring system, and climate variables (the cumulative rainfall and the mean temperature for May to October in each year). Township-level controls include farm-gate livestock prices, hay prices, wages for non-pastoral employment, and grassland rental prices. The reference year is 2010, the last year in our data set before GECP implementation. Standard errors are clustered by village and year. A two-sided t test is performed for each coefficient. Exact p-values are in parentheses.

*$p < 0.10$, **$p < 0.05$, ***$p < 0.01$

Supplementary Table 5. Summary statistics for control variables in all counties, separately for control and treatment groups

	All counties					
	Control group (5 N-NE provinces)			Treatment group (5 N-NW provinces)		
Variable	Obs.	Mean	Std. Dev.	Obs.	Mean	Std. Dev.
Rainfall (mm):						
May 2010	279	68.4	32.0	286	47.5	31.1
June 2010	279	36.5	21.6	286	36.0	29.5
July 2010	279	136.7	79.8	286	57.4	52.4
August 2010	279	177.7	80.3	286	54.4	46.1
September 2010	279	64.0	33.7	286	43.4	30.5
October 2010	279	31.3	17.6	286	24.8	15.0
Average temperature (°C)						
May 2010	279	16.3	2.5	286	13.9	3.9
June 2010	279	22.6	1.9	286	19.0	4.7
July 2010	279	24.1	2.3	286	21.6	4.3
August 2010	279	21.8	2.2	286	19.6	4.1
September 2010	279	16.8	2.4	286	14.7	3.5
October 2010	279	8.5	3.3	286	7.2	3.9
PSDI:						
May 2010	279	1.9	2.3	286	1.7	3.3
June 2010	279	-0.7	1.7	286	0.9	4.1
July 2010	279	-1.2	2.5	286	0.0	4.5
August 2010	279	-0.1	2.9	286	-0.6	4.4
September 2010	279	0.0	2.8	286	0.0	4.5
October 2010	279	0.1	2.8	286	0.8	4.5
Per-capita county fiscal income (yuan)	279	1221.6	1013.9	286	1806.7	3510.4

Supplementary Table 6. Summary statistics for control variables in all counties in border provinces, separately for control and treatment groups

	All counties in border provinces					
	Control group (5 N-NE provinces)			Treatment group (Inner Mongolia)		
Variable	Obs.	Mean	Std. Dev.	Obs.	Mean	Std. Dev.
Rainfall (mm):						
May 2010	279	68.4	32.0	83	59.5	28.5
June 2010	279	36.5	21.6	83	23.6	27.0
July 2010	279	136.7	79.8	83	60.6	53.7
August 2010	279	177.7	80.3	83	66.5	36.5
September 2010	279	64.0	33.7	83	54.4	34.5
October 2010	279	31.3	17.6	83	30.0	19.8
Average temperature (℃)						
May 2010	279	16.3	2.5	83	14.0	2.1
June 2010	279	22.6	1.9	83	21.2	1.9
July 2010	279	24.1	2.3	83	23.4	2.3
August 2010	279	21.8	2.2	83	19.5	2.5
September 2010	279	16.8	2.4	83	14.5	2.5
October 2010	279	8.5	3.3	83	5.4	3.3
PSDI:						
May 2010	279	1.9	2.3	83	2.5	1.8
June 2010	279	-0.7	1.7	83	-1.0	1.5
July 2010	279	-1.2	2.5	83	-2.9	1.8
August 2010	279	-0.1	2.9	83	-3.4	2.1
September 2010	279	0.0	2.8	83	-1.6	3.0
October 2010	279	0.1	2.8	83	0.0	2.8
Per-capita county fiscal income (yuan)	279	1221.6	1013.9	83	3352.5	5231.0

Supplementary Table 7. Summary statistics for control variables in border counties, separately for control and treatment groups

	Border counties					
	Control group (5 N-NE provinces)			Treatment group (Inner Mongolia)		
Variable	Obs.	Mean	Std. Dev.	Obs.	Mean	Std. Dev.
Rainfall (mm):						
May 2010	35	70.6	26.3	31	66.9	21.2
June 2010	35	28.8	9.8	31	23.4	8.3
July 2010	35	128.3	78.1	31	95.2	59.6
August 2010	35	118.4	50.0	31	91.7	33.2
September 2010	35	63.1	39.3	31	55.0	38.0
October 2010	35	41.1	19.2	31	35.9	18.4
Average temperature (℃)						
May 2010	35	14.8	1.8	31	13.5	2.0
June 2010	35	21.6	2.2	31	20.8	2.2
July 2010	35	23.2	1.5	31	22.3	1.9
August 2010	35	20.2	2.1	31	19.0	2.4
September 2010	35	15.2	2.2	31	13.9	2.5
October 2010	35	6.0	2.5	31	4.5	2.8
PSDI:						
May 2010	35	2.5	2.3	31	2.5	2.1
June 2010	35	-0.9	1.9	31	-1.4	1.3
July 2010	35	-1.3	2.9	31	-2.5	2.2
August 2010	35	-1.2	3.5	31	-2.7	2.6
September 2010	35	0.1	3.2	31	-1.0	3.1
October 2010	35	1.5	3.1	31	0.9	2.8
Per-capita county fiscal income (yuan)	35	886.9	671.6	31	1372.0	3288.1

Supplementary Table 8. Descriptive statistics of the key variables by year: household level

Year	Mean	Std.	Min	Max
Household level				
NDVI	0.562	0.212	0.027	1.000
Total year-end livestock (sheep unit)	306.53	278.35	0	2866.75
Cattle	160.86	178.81	0	1961.50
Sheep	124.58	184.31	0	1776.00
Supplementary feeding (kg/sheep unit)	37.09	98.10	0	1901.67
Grassland rent in or not	0.140	0.347	0	1
Annual household income (thousand yuan per capita)	18.03	33.47	-156.7	638.2
Net pastoral income per capita	12.53	30.44	-161.0	630.7
Non-pastoral income per capita	2.11	5.323	0	80000
GECP payment intensity (yuan/ha)	66.01	131.9	0	2547.3
Operated farm size (100 ha)	3.900	9.511	0	95.93
Number of laborers in raising livestock	2.011	1.298	0	8
Number of grassland plots	2.007	1.375	0	8
Share of joint operated area	0.165	0.343	0	1
Grass harvest indicator	0.156	0.363	0	1
Crop/fodder indicator	0.242	0.429	0	1
Education of the laborers (years)	5.109	3.590	0	16.5
Distance to the closest township-level road (km)	14.54	28.84	0	300
Village level				
Whether a village has local grassroots measures to limit grazing intensity (1 = yes, 0 = no)	0.634	0.482	0	1
Whether a village has a formal government-run monitoring system on grazing intensity (1 = yes, 0 = no)	0.519	0.500	0	1
Cumulative rainfall from May to September (mm)	264.21	154.70	2.3	604.6
Mean temperature from May to September (℃)	11.36	4.68	3.32	21.64
Township level				
Farm-gate livestock price (yuan/kg)				
Cattle	20.02	7.72	2.25	65.95
Sheep	14.60	7.26	3.28	32.52
Hay price (yuan/kg)	1.50	1.03	0.17	4.46
Wage for non-pastoral employment (1000 yuan/month)	2.34	0.76	0.61	4.58
Grassland rental price (yuan/ha)	161.64	221.48	0	827.41

Note: All price-related variables are deflated to the 2017 constant price.

Supplementary Table 9. Estimated impacts of GECP on herder income: household level

	(1)	(2)	(3)	(4)	(5)	(6)
	Household income per capita		Net pastoral income per capita		Non-pastoral income per capita	
	FE	RE	FE	RE	FE	RE
Annual GECP payment (yuan)	0.367***	0.446***	0.132*	0.261***	0.048	0.074***
	(0.000)	(0.000)	(0.080)	(0.000)	(0.224)	(0.008)
Control variables	Yes	Yes	Yes	Yes	Yes	Yes
Year fixed effects	Yes	Yes	Yes	Yes	Yes	Yes
Household fixed effects	Yes	No	Yes	No	Yes	No
Number of observations	3,474	3,474	3,474	3,474	3,474	3,474

Note: All outcome variables and the key explanatory variable, annual GECP payment, are transformed using an inverse hyperbolic sine transformation to avoid taking logarithm of zeros, following $\ln(y+(y^2+1)^{1/2})$. Household income includes net pastoral income, non-pastoral income, and GECP payment. Household-, village-, and township-level time-variant variables are controlled for. Household-level controls include quantity of labor used in raising livestock, operated farm size, share of joint operated area, total number of different plots, a dummy variable for grassland harvesting, and a dummy variable for planting crop/fodder. Village-level controls include an indicator of whether a village has local grassroots measures in place to limit grazing intensity, an indicator of whether a village has a formal government-run monitoring system, and climate variables (the cumulative rainfall and the mean temperature from May to October in each year). Township-level controls include farm-gate livestock prices, hay prices, wages for non-pastoral employment, and grassland rental prices. Standard errors are clustered by village and year. A two-sided *t* test is performed for each coefficient. Exact *p*-values are in parentheses.

*$p < 0.10$, **$p < 0.05$, ***$p < 0.01$
